# Screening of lipids and kidney function in children and adolescents with Type 1 Diabetes: does age matter?

**DOI:** 10.3389/fendo.2023.1186913

**Published:** 2023-06-02

**Authors:** Eulalia Catamo, Antonietta Robino, Klemen Dovc, Davide Tinti, Gianluca Tamaro, Riccardo Bonfanti, Roberto Franceschi, Ivana Rabbone, Tadej Battelino, Gianluca Tornese

**Affiliations:** ^1^ Institute for Maternal and Child Health, Istituto di Ricovero e Cura a Carattere Scientifico (IRCCS) Burlo Garofolo, Trieste, Italy; ^2^ Faculty of Medicine, University of Ljubljana, Ljubljana, Slovenia; ^3^ Department of Endocrinology, Diabetes and Metabolism, University Children’s Hospital, University Medical Centre Ljubljana, Ljubljana, Slovenia; ^4^ Center for Pediatric Diabetology, Azienda Ospedaliero-Universitaria (AOU) Città Della Salute e Della Scienza, Torino, Italy; ^5^ Diabetes Research Institute, Department of Pediatrics, IRCCS San Raffaele Hospital, Milano, Italy; ^6^ Division of Pediatrics, S. Chiara General Hospital, Trento, Italy; ^7^ Division of Pediatrics, Department of Health Sciences, Università Del Piemonte Orientale, Novara, Italy

**Keywords:** type 1 diabetes, lipids profile, kidney function, age, guidelines

## Abstract

**Introduction:**

The purpose of this study was to evaluate lipid profile and kidney function in children and adolescents with Type 1 Diabetes.

**Methods:**

This was a retrospective study including 324 children and adolescents with Type 1 Diabetes (48% females, mean age 13.1 ± 3.2 years). For all participants, demographic and clinical information were collected. The prevalence of dyslipidemia and kidney function markers were analyzed according to age. Multivariate linear regression analyses were performed to test the association of lipids or markers of renal function with demographic and clinical information (sex, age, disease duration, BMI SDS, HbA1c).

**Results:**

In our study the rate of dyslipidemia reached 32% in children <11 years and 18.5% in those ≥11 years. Children <11 years presented significantly higher triglyceride values. While the albumin-to-creatinine ratio was normal in all individuals, 17% had mildly reduced estimated glomerular filtration rate. Median of HbA1c was the most important determinant of lipids and kidney function, being associated with Total Cholesterol (p-value<0.001); LDL Cholesterol (p-value=0.009), HDL Cholesterol (p-value=0.045) and eGFR (p-value=0.001).

**Conclusion:**

Dyslipidemia could be present both in children and adolescents, suggesting that screening for markers of diabetic complications should be performed regardless of age, pubertal stage, or disease duration, to optimize glycemia and medical nutrition therapy and/or to start a specific medical treatment.

## 1 Introduction

The development of micro and macrovascular complications is rare in children and adolescents with Type 1 Diabetes (T1D) meeting recommended HbA1c value: a declining incidence of vascular complications has been reported in the developed world (1, 2), while it is still an issue in developing countries (3).

The 2023 ADA Standards of Medical Care and the 2022 ISPAD Clinical Practice in children and adolescents with T1D (4, 5) still suggest an age limit to perform screening for risk factors for diabetes-related complications, such as dyslipidemia and albuminuria. Nevertheless, several studies demonstrated that the atherosclerotic process begins in childhood and that the first subclinical indications of cardiovascular risk may occur before puberty (6). Lipids above the recommended values were previously reported in subjects with T1D from 2 years of age, confirming that risk factors for cardiovascular complications start early after diabetes diagnosis (7, 8). Similarly, decreased estimated glomerular filtration rates were reported in children with T1D between 2 and 18 years of age (9).

However, overall, these past studies did not perform a comparison of the prevalence of lipid and renal abnormalities among T1D subjects belonging to different age groups.

Therefore, in this study, we analyzed lipid profile and kidney function in children and adolescents with T1D stratified for cut-off established by ADA and ISPAD guidelines for start to screening, hypothesizing that their screening is of fundamental importance, regardless of age, pubertal status and disease duration. Moreover, we also analyzed demographic and clinical factors associated with lipids and renal markers.

## 2 Methods

### 2.1 Study participants

Three hundred twenty-four subjects with T1D and age <21 years, with at least one year of disease duration, were recruited at Diabetes Units of IRCCS Burlo Garofolo (Trieste, Italy) (n=90), Regina Margherita Children’s hospital (Torino, Italy) (n=90), Santa Chiara hospital (Trento, Italy), (n=14), University Medical Center (Ljubljana, Slovenia) (n=80) and IRCCS San Raffaele (Milano, Italy) (n=50) between 2018 and 2021.

### 2.2 Study procedures

We retrospectively collected demographic and anthropometric information, such as age, sex, height, weight, and pubertal status. Puberty is defined as the presence of breast budding in girls and a testicular volume of 4 ml in boys. Standard deviation scores of body mass index (BMI SDS) were calculated according to WHO reference charts using the Growth Calculator 4 software (http://www.weboriented.it/gh4/).

Clinical information included age at diagnosis, disease duration, and HbA1c at the regular visits in the previous year. Based on the cut-off of HbA1c of 7% (53mmol/mol), T1D subjects were categorized in two groups (4, 5).

Laboratory data were collected from the latest performed screening.

Fasting lipids were collected, and the lipids profile was defined as acceptable, borderline, and abnormal according to the NHLBI guidelines (10). The following cut-off points for dyslipidemia definition were considered: total cholesterol (TC) ≥200 mg/dL; LDL cholesterol (LDL-C) ≥130 mg/dL; HDL cholesterol (HDL-C) <40 mg/dL; triglycerides (TG) ≥100 mg/dL up to 9 years and triglycerides ≥130 mg/dL over 9 years. Subjects with single or combined lipids alterations were considered “abnormal lipid profile.”

Evaluation of albuminuria was determined as albumin to creatinine ratio (ACR), by first monitoring urine sample, and ACR value >30 mg/g were considered abnormal (11).

The estimated glomerular filtration rate (eGFR) was calculated using Schwartz’s equation: 0.413 x height (cm)/serum creatinine mg/dL (12). eGFR values <90 mL/min/1.73 m^2^ were considered abnormal (11).

### 2.3 Statistical analysis

Descriptive statistics represent percentages, means, standard deviations (SD). T1D subjects were stratified for pubertal status (pre-pubertal *vs.* pubertal), disease duration (<5 *vs.* ≥5 years) following ADA or ISPAD guidelines. Moreover, for age ISPAD guidelines (<11 *vs.* ≥11) were applied for lipids analysis, while ADA guidelines (≤10 *vs.* >10 years) for kidney function analysis.

The skewness and kurtosis were calculated for testing the normality. Differences between T1D individuals stratified for age, pubertal status or disease duration were analyzed by chi-squared tests to compare categorical data. While t-tests or Mann-Whitney test used to compare the means when the variable is normally or not-normally distributed, respectively.

Multivariate linear regression analyses were performed to test the association of lipids or markers of renal function with sex, age, disease duration, BMI SDS and median HbA1c as explanatory variables.

Statistical analyses were performed with R stats package, v 4.2.2 (www.r-project.org).

## 3 Results

Among the 324 T1D individuals included in this study, 156 (48%) were females. The mean age at enrollment was 13.1 ± 3.2 years, and 77% were pubertal, with a mean BMI SDS of 0.13 ± 1.1. The mean disease duration was 5.4 ± 3.6 years. Median HbA1c over the previous year was 7.69 ± 1.01% (60 mmol/mol). T1D characteristics were summarized in **Table 1**.

**Table 1:** Demographic, anthropometric and clinical information in T1D subjects stratified by age.

	All (n=324)	<11 years (n=93)	≥11 years (n=231)	p-value
Age mean ± sd	13.1 ± 3.2	9.03 ± 1.5	14.7 ± 2.1	**<0.001**
Sex				**0.03**
females (%)	48%	58%	44%	
males (%)	52%	42%	56%	
Standardized BMI mean ± sd	0.14 ± 1.1	-0.06 ± 1.1	0.21 ± 1.1	**0.04**
HbA1c mean ± sd	7.69 ± 1.01	7.59 ± 0.95	7.72 ± 1.03	0.26
HbA1c ≥7% (≥53 mmol/mol)	75%	69%	77%	**0.15**
Age at onset mean ± sd	7.7 ± 3.9	5.2 ± 2.4	8.7 ± 3.8	**<0.001**
Years of disease mean ± sd	5.4 ± 3.6	3.9 ± 2.4	6.0 ± .3.8	**<0.001**
Puberty yes (%)	76%	29.5%	99%	**<0.001**

Significant results were indicated in bold (p-value<0.05).

T1D subjects are stratified according to the ADA or ISPAD guidelines.

Differences by age were computed by t-test and chi-square test.

Lipid profiles and renal function markers are reported in **Table 2** and **Table 3**, respectively.

**Table 2:** Lipid profile in T1D subjects stratified by age.

	All (n=324)	<11 years (n=93)	≥11 years (n=231)	p-value
**TC** (mg/dL) mean ± sd	164.7 ± 28.8	165.9 ± 26.4	164.2 ± 29.8	0.62
Acceptable	60%	58%	61%	0.68
Borderline	30%	33%	28%	
Abnormal	10%	9%	11%	
**LDL-C** (mg/dL) mean ± sd	87.9 ± 25.5	85.9 ± 23.3	88.8 ± 26.4	0.35
Acceptable	83%	86%	82%	0.30
Borderline	10%	10.5%	10%	
Abnormal	7%	3.5%	8%	
**HDL-C** (mg/dL) mean ± sd	62.4 ± 15.3	65.4 ± 17.2	61.2 ± 14.3	**0.047**
Acceptable	89%	92%	87%	0.10
Borderline	7%	2%	9%	
Abnormal	4%	6%	4%	
**TG** (mg/dL) mean ± sd	72.8 ± 37.0	76.05 ± 38.7	71.4 ± 36.4	0.33
Acceptable	77%	72%	79%	**0.01**
Borderline	14%	12%	15%	
Abnormal	9%	16%	6%	
Lipid profile				
Acceptable	43%	42%	45%	0.15
Borderline	36%	26%	36.5%	
Abnormal	21%	32%	18.5%	

Significant results were indicated in bold (p-value<0.05).

T1D subjects are stratified according to the ADA or ISPAD guidelines.

Differences by age were computed by t-test, Mann-Whitney U test and chi-square test.

HDL-C, HDL cholesterol; LDL-C, LDL cholesterol; TC, total cholesterol; TG, triglycerides.

Lipid profile was defined as acceptable, borderline, and abnormal according to the NHLBI guidelines (7).

**Table 3:** Kidney function markers in T1D subjects stratified by age, pubertal status and disease duration.

	All (n=324)	Age ≤10y (n=63)	Age >10y (n=261)	p-value	Pre-pubertal (n=64)	Pubertal (n=210)	p-value	Disease duration <5y (n=175)	Disease duration ≥5y (n=149)	p-value
**ACR** (mg/gr) mean ± sd	4.0 ± 5.7	4.6 ± 6.4	3.9 ± 5.6	0.56	6.3 ± 8.0	3.4 ± 5.1	0.02	4.5 ± 5.9	3.5 ± 5.6	0.18
**eGFR** (mL/min/1.73 m^2^) mean ± sd	114.2 ± 33.7	113.2 ± 38.1	114.4 ± 32.8	0.59	113.4 ± 34.9	115.7 ± 33.2	0.94	113.6 ± 33.9	114.8 ± 33.6	0.89
**eGFR <**90 (mL/min/1.73 m^2^) yes (%)	17%	25%	15%	0.26	23%	14%	0.25	19.5%	14%	0.42

Differences were computed by Mann-Whitney U test and chi-square test.

T1D subjects are stratified according to the ADA or ISPAD guidelines.

ACR, urinary albumin-to-creatinine ratio; eGFR, estimated glomerular filtration rate.

Overall, 21% (n=65) of the participants had abnormal plasma lipid values; the percentage of dyslipidemia reached 32% (n=25) in children <11 and 18.5% (n=40) in those ≥11 years. Moreover, TG values were higher in individuals <11 (n=14) than in those ≥11 years (n=12) (16% *vs.* 6%, p-value=0.01) (**Table 2**). No other significant differences in lipid levels emerged, except for HDL-C values, which were slightly higher in children <11 than in children ≥11 years (p-value=0.047).

No significant differences emerged for eGFR. ACR levels are statistically different between non-pubertal and pubertal T1D subjects (p value=0.02), although the ACR values are in the normal range in both groups. Furthermore, around 17% of T1D subjects presented an eGFR <90 mL/min/1.73 m^2^, and this percentage reached 25% in children aged ≤10 years (**Table 3**).

At multivariate analysis, higher TC levels were associated with female sex (p-value=0.001) and high median HbA1c over the previous year (p-value<0.001); higher LDL-C levels were associated with increasing age (p-value=0.007), female sex (p-value=0.009), high median HbA1c (p-value=0.009) and slightly with increased BMI SDS (p-value=0.046); higher HDL-C value was associated with younger age (p-value=0.001) and high median HbA1c (p-value=0.045); finally, higher TG levels were directly associated with increased median HbA1c (p-value=0.033) (**Table 4**).

**Table 4:** Factors associated with lipids and renal function as assessed by multivariate regression models.

	Lipids				Renal function		
**Explanatory variables**	**Total Cholesterol** (mg/dL)	**LDL Cholesterol** (mg/dL)	**HDL Cholesterol** (mg/dL)	**Triglycerides** (mg/dL)	**ACR** (mg/gr)	**eGFR** (mL/min/1.73 m^2^)	**eGFR <**90 (mL/min/1.73 m^2^) % yes
*Age*	0.41 (0.44)	**0.007 (1.29)**	**0.001 (-0.95)**	0.72 (-0.25)	0.69 (0.06)	0.46 (-0.59)	0.59 (-0.94)
*Sex*	**0.001 (-10.5)**	**0.009 (-7.61)**	0.46 (-1.30)	0.22 (-5.27)	**0.017 (-2.4)**	**0.02 (-11.1)**	**0.009 (0.06)**
*Years of disease*	0.61 (-0.25)	0.36 (-0.40)	0.33 (0.26)	0.44 (0.50)	0.62 (-0.07)	0.79 (-0.19)	0.56 (-0.03)
*Standardized BMI*	0.11 (2.34)	**0.046 (2.65)**	0.64 (-0.38)	0.22 (2.44)	0.26 (-0.49)	0.21 (-2.70)	**0.045 (0.35)**
*Median HbA1c*	**<0.001 (6.8)**	**0.009 (3.82)**	**0.045 (1.80)**	**0.033 (4.71)**	0.18 (-0.65)	**0.001 (7.42)**	0.14 (-0.30)

The values are p-value and beta in brackets.

Significant results were indicated in bold (p-values<0.05).

For the non-quantitative Sex variable, the reference category is female.

Moreover, greater ACR levels were associated with female sex (p-value=0.01), whereas higher eGFR levels were associated with female sex (p-value=0.02) and higher HbA1c value (p-value=0.001) (**Table 4**). Finally, eGFR<90 was associated with male sex (p-value=0.009) and with slightly increased BMI (p-value=0.045).

## 4 Discussion

Despite improvements in management, kidney and cardiovascular diseases are still significant causes of mortality among people with T1D (13, 14). Although they rarely occur during childhood, changes in cardiac function and lipid levels may indicate a disease process that begins early in the course of the disease (15, 16); moreover, disease onset before the age of 15 has been associated with early onset albuminuria with a more rapid decrease in eGFR (17). Intensive education and treatment at a young age may prevent or delay the onset and progression of complications (18), with a positive ‘legacy effect’ even after 30 years (19). To prevent the development of such complications, diabetes management requires multi-level risk reduction strategies that include also screening for risk factors (20).

Concerning dyslipidemia screening, ADA recommended that “if initial LDL-C is <100 mg/dL, subsequent testing should be performed at 9–11 years of age” (4). Similarly, ISPAD recommends that “screening for dyslipidemia is recommended soon after diagnosis (when glycemia is stabilized) in all young people with T1D from age 11 years”, in the absence of a family history of hypercholesterolemia or early cardiovascular death (5).

In the present study, we showed that not only there are no significant differences in lipids profile in children below and over 11 years, but also that rate of dyslipidemia (defined as single or combined lipids alterations) reached 32% in children <11 years compared to 18.5% in those ≥11 years.

We also found that elevated lipid levels were associated with higher HbA1c, confirming HbA1c over the previous year as one of the most important determinants of lipid profile. Moreover, LDL-C and HDL-C levels were associated with age, with a direct and inverse relationship, respectively. With regards to HDL-C (known as the “good” cholesterol for the well-documented inverse relationship with adverse cardiovascular outcomes), it has been shown that, in the presence of chronic inflammation or renal dysfunction, it might reverse its effect and become detrimental to endothelial function (21). Interestingly, children <11 years presented higher triglyceride values than adolescents (p-value=0.01), although this age group had fewer non-modifiable risk factors for diabetes mismanagement, such as puberty and long disease duration.

With regards to nephropathy screening, ADA recommended that “annual screening for albuminuria with a random spot urine sample for ACR should be considered at puberty or at age >10 years, whichever is earlier, once the child has had diabetes for five years” and that “eGFR should be considered at baseline and repeated as indicated based on clinical status, age, diabetes duration, and therapies” (4). Similar recommendations are given in the ISPAD guidelines that recommend starting screening for ACR and eGFR at puberty or age 11 years with 2-5 years of diabetes duration (5). In our sample, ACR was normal in all individuals. However, it has been proposed that non-albuminuric patients with diabetes may progress in any case toward chronic kidney disease; as a matter of fact, in a previous study also pediatric individuals with normal albuminuria but mildly reduced eGFR (60-89 mL/min/1.73 m^2^) showed a worst cardiometabolic risk profile with higher levels of insulin requirement, TG/HDL-C ratio, neutrophil/lymphocytes ratio, blood pressure, uric acid, and low HDL-C levels (22). The rate of subjects with an eGFR value below 90 mL/min/1.73 m^2^ in the present study was 17%, and this figure reached 25% in children under the age of 10 and 23% in the prepubertal stage, although there were no statistically significant differences between age, pubertal status, or disease duration. On the contrary, higher eGFR was associated with higher HbA1c, that directly correlates with hyperfiltration, also in individuals with short duration of T1D (23).

## 5 Limitations of the study

A potential limitation of this study is the lack of information on LDL-C levels at onset and family history. In addition, we have no information on other markers for the screening of renal function, such as Cystatin C. Lastly, the recruitment of only European samples, makes our findings not generalizable to other ethnic groups. Despite these limitations, the accuracy of collected clinical and demographic information is the key strength of the present study.

## 6 Conclusions

The findings of our study show that diabetes-associated risk factors, such as abnormal lipid profiles and mildly reduced eGFR, are already present in childhood, suggesting that screening for markers of diabetic complications should be performed regardless of age, pubertal stage, or disease duration, to optimize glycemia and medical nutrition therapy and/or to start a specific medical treatment. This should be considered in clinical management and the following standards of medical care.

## Data availability statement

The raw data supporting the conclusions of this article will be made available by the authors, without undue reservation.

## Ethics statement

The ethics committee approved the study [CEUR-2018-Em-323-Burlo (Italy) and KME-0120-65/2019/4]. Written informed consent to participate in this study was provided by the participants’ legal guardian/next of kin.

## Author contributions

EC, AR, and GTo designed and performed the research. EC, KD, DT, GTa, RB, RF acquired data. EC and AR analyzed and interpreted data. EC, AR, and GTo drafted the manuscript. EC, AR, KD, DT, GTa, RB, RF, IR, TB, and GTo revised the manuscript. GTo is the guarantor of this work and, as such, has full access to all the data in the study and takes responsibility for the integrity of the data and the accuracy of the data analysis. All authors contributed to the article and approved the submitted version.

## References

[1] Bojestig M Arnqvist HJ Hermansson G Karlberg BE Ludvigsson J. Declining incidence of nephropathy in insulin-dependent diabetes mellitus. N Engl J Med (1994) 330(1):15–8. doi: 10.1056/nejm199401063300103 8259139

[2] Downie E Craig ME Hing S Cusumano J Chan AK Donaghue KC. Continued reduction in the prevalence of retinopathy in adolescents with type 1 diabetes: role of insulin therapy and glycemic control. Diabetes Care (2011) 34(11):2368–73. doi: 10.2337/dc11-0102 PMC3198305 22025782

[3] Majaliwa ES Munubhi E Ramaiya K Mpembeni R Sanyiwa A Mohn A. Survey on acute and chronic complications in children and adolescents with type 1 diabetes at muhimbili national hospital in dar es salaam, Tanzania. Diabetes Care (2007) 30(9):2187–92. doi: 10.2337/dc07-0594 17563337

[4] ElSayed NA Aleppo G Aroda VR Bannuru RR Brown FM Bruemmer D. 14. children and adolescent: standard of care in diabetes-2023. Diabetes Care (2023) 146(Suppl 1):S230–53. doi: 10.2337/dc23-S014 PMC9810473 36507640

[5] Bjornstad P Dart A Donaghue KC Dost A Feldman EL Tan GS. ISPAD clinical practice consensus guidelines 2022: microvascular and macrovascular complications in children and adolescents with diabetes. Pediatr Diabetes (2022) 23(8):1432–50. doi: 10.1111/pedi.13444 36537531

[6] Haller MJ Stein J Shuster J Theriaque D Silverstein J Schatz DA. Peripheral artery tonometry demonstrates altered endothelial function in children with type 1 diabetes. Pediatr Diabetes (2007) 8(4):193–8. doi: 10.1111/j.1399-5448.2007.00246.x 17659060

[7] Fornari E Piona C Rabbone I Cardella F Mozzillo E Predieri B. Cardiovascular risk factors in children and adolescents with type 1 diabetes in Italy: a multicentric observational study. Pediatr Diabetes (2020) 21(8):1546–55. doi: 10.1111/pedi.13123 32939906

[8] Schwab KO Doerfer J Hecker W Grulich-Henn J Wiemann D Kordonouri O. Spectrum and prevalence of atherogenic risk factors in 27,358 children, adolescents, and young adults with type 1 diabetes: cross-sectional data from the German diabetes documentation and quality management system (DPV). Diabetes Care (2006) 29(2):218–25. doi: 10.2337/diacare.29.02.06.dc05-0724 16443863

[9] Favel K Irvine M Ronsley R Panagiotopoulos C Mammen C. Glomerular filtration rate abnormalities in children with type 1 diabetes. Can J Diabetes (2022) 46(5):457–63.e1. doi: 10.1016/j.jcjd.2022.01.007 35752567

[10] Expert Panel of Integrated Guidelines for Cardiovascular Health and Risk Reduction in Children and Adolescent; National Heart, Lung and Blood Institute. Expert panel on integrated guidelines for cardiovascular health and risk reduction in children and adolescents: summary report. Pediatrics (2011) 128(5):S213–56. doi: 10.1542/peds.2009-2107C PMC4536582 22084329

[11] Levey AS Eckardt K-U Dorman NM Christiansen SL Hoorn EJ Ingelfinger JR. Nomenclature for kidney function and disease: report of a kidney disease: improving global outcomes (KDIGO) consensus conference. Kidney Int (2020) 97(6):1117–29. doi: 10.1016/j.kint.2020.02.010 32409237

[12] Schwartz GJ Muñoz A Schneider MF Mak RH Kaskel F Warady BA. New equations to estimate GFR in children with CKD. J Am Soc Nephrol (2009) 20(3):629–37. doi: 10.1681/ASN.2008030287 PMC2653687 19158356

[13] Groop PH Thomas MC Moran JL Wadèn J Thorn LM Mäkinen VP. The presence and severity of chronic kidney disease predicts all-cause mortality in type 1 diabetes. Diabetes (2009) 58(7):1651–8. doi: 10.2337/db08-1543 PMC2699848 19401416

[14] Sharma H Lencioni M Narendran P. Cardiovascular disease in type 1 diabetes. Cardiovasc Endocrinol Metab (2019) 8(1):28–34. doi: 10.1097/XCE.0000000000000167 31646295 PMC6739891

[15] Hensel KO Grimmer F Roskopf M Jenke AC Wirth S Heusch A. Subclinical alterations of cardiac mechanics present early in the course of pediatric type 1 diabetes mellitus: a prospective blinded speckle tracking stress echocardiography study. J Diabetes Res (2016) 2016:2583747. doi: 10.1155/2016/2583747 26839891 PMC4709644

[16] Maahs DM Dabelea D D’Agostino RB Jr Andrews JS Shah AS Crimmins N. Glucose control predicts 2-year change in lipid profile in youth with type 1 diabetes. J Pediatr (2013) 162(1):101–7.e1. doi: 10.1016/j.jpeds.2012.06.006 22795314 PMC3807690

[17] Edqvist J Rawshani A Rawshani A Adiels M Franzén S Bjorck L. Trajectories in HbA1c and other risk factors among adults with type 1 diabetes by age at onset. BMJ Open Diabetes Res Care (2021) 9(1):e002187. doi: 10.1136/bmjdrc-2021-002187 PMC8169495 34059526

[18] Diabetes Control and Complications Trial Research Group. Effect of intensive diabetes treatment on developing and progressing long-term complications in adolescents with insulin-dependent diabetes mellitus: diabetes control and complications trial. J Pediatr (1994) 125(2):177–88. doi: 10.1016/s0022-3476(94)70190-3 8040759

[19] Diabetes Control and Complications Trial (DCCT)/Epidemiology of Diabetes Interventions and Complications (EDIC) Study Research Group. Intensive diabetes treatment and cardiovascular outcomes in type 1 diabetes: the DCCT/EDIC study 30-year follow-up. Diabetes Care (2016) 39(5):686–93. doi: 10.2337/dc15-1990 PMC4839174 26861924

[20] Lithovius R Harjutsalo V Forsblom C Saraheimo M Groop PH. The consequences of failure to achieve targets of guidelines for prevention and treatment of diabetic complications in patients with type 1 diabetes. Acta Diabetol (2015) 52(1):31–8. doi: 10.1007/s00592-014-0595-x 24849006

[21] Chiesa ST Charakida M McLoughlin E Nguyen HC Georgiopoulos G Motran L. Elevated high-density lipoprotein in adolescents with type 1 diabetes is associated with endothelial dysfunction in the presence of systemic inflammation. Eur Heart J (2019) 40(43):3559–66. doi: 10.1093/eurheartj/ehz114 PMC6855140 30863865

[22] Di Bonito P Mozzillo E Rosanio FM Maltoni G Piona CA Franceschi R. Albuminuric and non-albuminuric reduced eGFR phenotypes in youth with type 1 diabetes: factors associated with cardiometabolic risk. Nutr Metab Cardiovasc Dis (2021) 31(7):2033–41. doi: 10.1016/j.numecd.2021.03.019 34083127

[23] Soper CP Barron JL Hyer SL. Long-term glycaemic control directly correlates with glomerular filtration rate in early type 1 diabetes mellitus before the onset of microalbuminuria. Diabetes Med (1998) 15(12):1010–4. doi: 10.1002/(SICI)1096-9136(1998120)15:12<1010:AID-DIA703>3.0.CO;2-H 9868973

